# Facility and home based HIV Counseling and Testing: a comparative analysis of uptake of services by rural communities in southwestern Uganda

**DOI:** 10.1186/1472-6963-11-54

**Published:** 2011-03-04

**Authors:** Edgar M Mulogo, Aden S Abdulaziz, Ranieri Guerra, Sebastian O Baine

**Affiliations:** 1 Department of Community Health, Mbarara University of Science and Technology, PO Box 1410, Mbarara, Uganda; 2 Istituto Superiore di Sanita, Istituto Superiore di Sanita, Via Giano della Bella, 34, 00162 Rome, Italy; 3 Makerere University School of Public Health, Makerere University Kampala, PO Box 7072, Kampala, Uganda

## Abstract

**Background:**

In Uganda, public human immunodeficiency virus (HIV) Voluntary Counseling and Testing (VCT) services are mainly provided through the facility based model, although the home based approach is being promoted as a strategy for improving access to VCT. However the uptake of VCT varies according to service delivery model and is influenced by a number of factors. The aim of this study therefore, was to compare predictors for uptake of facility and home based VCT in a rural context.

**Methods:**

A longitudinal study with cross-sectional investigative phases was conducted at two sites (Rugando and Kabingo) in southwestern Uganda between November 2007 (baseline) and March 2008 (follow up). During the baseline visit, facility based VCT was offered at the main health centre in Rugando while home based VCT was offered at the household level in Kabingo and a mixed survey questionnaire administered to the respondents. The results presented in this paper are derived from only the baseline data.

**Results:**

Nine hundred ninety four (994) respondents were interviewed, of whom 500 received facility based VCT in Rugando and 494 home based VCT in Kabingo during the baseline visit. The respondents had a mean age of 32.2 years (SD 10.9) and were mainly female (68 percent). Clients who received facility based VCT were less likely to be residents of the more rural households (adjusted Odds Ratio (aOR) = 0.14, 95% CI 0.07, 0.22). The clients who received home based VCT were less likely to report having an STI symptom (aOR = 0.63, 95% CI 0.46, 0.86), and more likely to be worried about discrimination if they contracted AIDS (aOR = 1.78, 95% CI 1.22, 2.61).

**Conclusion:**

The uptake of VCT provided through either the facility or home based models is influenced by client characteristics such as proximity to service delivery points, HIV related symptoms, and fear of discrimination in rural Uganda. Interventions that seek to improve uptake of VCT should provide potential clients with both facility and home based VCT options within a given setting. The clients are then able to select a model for VCT that best fits their characteristics. This is likely to have positive implications for both service coverage and uptake by different sub-groups within particular communities.

## Background

The Joint United Nations Programme on HIV/AIDS (UNAIDS) and World Health Organization (WHO) estimate that the number of people living with HIV or Acquired Immune Defiency Syndrome (AIDS) in 2008 stood at 33 million [1]. Although sub-Saharan Africa is comprised of only 10% of the world's population, it is home to 67% of all people living with HIV - some 22 million [1]. In 2007 an estimated 2.7 million people were newly infected [1]. Declines in adult national prevalence appear to be underway in three sub-Saharan African countries: Kenya, Uganda and Zimbabwe [2].

Uganda has made remarkable progress in reversing the trend of the HIV/AIDS epidemic in the last two decades. The HIV/AIDS Sero-Behavioral Survey (UHSBS) conducted in 2004/2005 showed a national adult HIV prevalence of 6.4% [3] for the 15-49 age group, down from a peak of 28% in 1992 [4]. This achievement has largely been attributed to the ABC strategy (abstinence, be faithful, condom use), treatment of opportunistic infections, prevention of mother-to child transmission of HIV (PMTCT), use of antiretroviral therapy (ART) and HIV counseling and testing [5]. Recent evidence however suggests that the incidence of HIV is rising among some population sub-groups in Uganda [6].

HIV Voluntary Counseling and Testing (VCT) is now a key component of national AIDS programmes in sub-Saharan Africa [7], and is a major component of HIV/AIDS prevention, treatment and care efforts in Uganda [8]. The UNAIDS in its guidelines on terminology reports that an alternative term for VCT is client initiated testing [9]. VCT relies mostly on individuals presenting themselves for HIV testing and giving voluntary informed consent [10]. It is a process whereby individuals or couples undergo pre-test counseling, risk assessment, a same-day rapid HIV test, post-test HIV prevention counseling, and referral for medical and support services by trained counselors [11]. Matovu et al report that post-test counseling gives high-risk HIV-uninfected individuals the opportunity to change their high-risk behavior and potentially, the behavior of their sex and drug-using partners [8]. As a result, expansion of VCT services has been advocated for, as a central component of public health efforts to bring down the HIV incidence through reduction in high-risk behaviors [8].

As access to anti-retroviral treatment is scaled up in countries like Uganda, there is a critical opportunity to simultaneously expand access to HIV prevention, which continues to be the mainstay of the response to the HIV epidemic [12]. The WHO and UNAIDS report that even in settings where antiretroviral therapy is not yet available, interventions such as co-trimoxazole prophylaxis and antiretroviral prophylaxis for the PMTCT offer significant potential health benefits to individuals and their children [13].

VCT can be offered through different innovative approaches such as in the work place, in mobile clinics and using the home based model [13]. VCT services in Uganda are provided through facility based services, stand-alone (freestanding) clinics and mobile outreach programmes. The provision of VCT through a home based approach was initiated in the country as a strategy for improving access to the services. In the facility based model, counseling and testing (CT) services are offered in medical settings (primarily the public sector). However, they may not appeal to groups who often do not go readily to health facilities, such as young people and men. Also, in the presence of competing interests, staff may not be committed to the VCT program [10]. In quasi-integrated sites, an NGO provides CT in a public sector health facility; both the NGO and the facility contribute to managing the services [14]. While in the home based model, CT is offered within the home to family members, including children where appropriate [14]. Home based VCT involves the use of lay counselors or community health workers to provide counseling and testing. The counselors and testers move from door to door, provide pre-test counseling, and take consent from eligible family members [10]. For this reason, it is sometimes referred to as the family-based model [14].

Coverage of client-initiated HIV testing and counseling services is inadequate in both high-income and resource-constrained settings [13]. It has been reported that in developing countries only one out of nine people has access to voluntary counseling and testing [15]. A survey conducted in Uganda in 2006 shows that the percentage of women and men aged 15-49 who received an HIV test in the last 12 months and who knew their results was 12% [16].

The uptake of VCT varies according to service delivery model and this is influenced by a number of factors. An annual serosurvey conducted in rural southwestern Uganda reports only 20% of participants had received their HIV results under the existing facility-based system [17]. In a study cohort in Rakai district, Uganda, uptake of results delivered at home rose from 35% in the first year of the study in 1994-5 to 65% in 1999-2000 [18]. Were et al also demonstrated that onetime home delivery of HIV results and counseling following community-wide mobilization in Mukono district of Uganda yielded a remarkable 93% uptake [19].

Some studies have found that acceptance of VCT programs has been poor due to socio-cultural barriers. In Nigeria, women reported reluctance to test because of the discrimination and stigma that is placed upon those who openly acknowledge their HIV status [20]. A study undertaken in Tanzania reported that socio-demographic factors such as gender and marital status influenced VCT uptake among males and females in different ways concurring partly with what had earlier been established by Matovu et al [7,21]. Elsewhere Pool et al report that confidentiality, social stigma and domestic violence reduced acceptance of VCT among rural Ugandan respondents [22]. Nyanzi-Wakholi et al reported that the decision to take up VCT was postponed until the person or their partner was evidently sick [23].

Studies undertaken on factors influencing uptake of VCT have mainly dwelt on specific models and there is paucity of literature comparing the facility and home based approaches. This study therefore seeks to compare predictors for uptake of facility and home based VCT in a rural context. Considering that the home based model is being promoted as a strategy for improving access, it is expected that findings from this analysis will provide a basis for a review of the design and implementation of existing models for VCT provision in rural communities of Uganda.

## Methods

### Design and sample population

A longitudinal study with cross-sectional investigative phases was conducted in two sub-counties (southwestern Uganda) between November 2007 (baseline) and March 2008 (follow up). The follow up visit was scheduled three months after the baseline because the national HIV testing policy recommends another test at 3 months. The two sites were Rugando and Kabingo sub-counties in Mbarara and Isingiro districts respectively. Mbarara and Isingiro districts have estimated populations of 418,300 and 385,500 respectively [24]. These districts were purposely chosen to represent "average" rural districts according to objective performance accomplishment scores (measuring the degree to which HIV/AIDS health-related practices and objectives are met at health facilities and communities within the districts e.g. utilization of out-patient services). In 2007 the number of health units in Mbarara were 53 and 49 in Isingiro [24]. While per capita out-patient department utilization between 2007 and 2008 in public and private not for profit health units was 0.8 for Mbarara and 0.9 for Isingiro [24].

During the baseline visit facility based VCT was offered at the main health centre (health centre level 4) in Rugando sub-county while home based VCT was offered at the household level in Kabingo. Prior to providing the VCT intervention in both sub-counties the service was promoted through the local community leaders and community health workers. At the Rugando health centre, clients were sampled consecutively from those willing to receive facility based VCT. In order to minimize loss to follow up, only clients residing within a 5 kilometer radius of the health facility were selected. In Kabingo sub-county, one parish was randomly selected and home based VCT offered in all the 9 villages within this parish. The number of households visited in each village was determined using the probability proportionate to size method. The households visited in each village were then randomly selected from a list of the households compiled by the local leader of the village. In each household an adult between 18 and 59 years was offered VCT. The required sample size was estimated using a formula reported by Hayes et al, and was based on a number of assumptions [25]. A study in Rakai showed an uptake of 50% while another study in Tororo showed an uptake of 90% [19,26]. To estimate sample size, an expected uptake of 70% was assumed for home based VCT. The level of uptake at the Rugando health facility was estimated at 60% based on figures for the antenatal clinic prevention of mother-to-child transmission (PMTCT) of HIV services. The PMTCT services are offered under routine outpatient settings in Ugandan health facilities and therefore are considered representative of a health facility based VCT services. Assuming a two sided type 1 error rate of 0.05 and a power of 90%, p_h _= level of uptake for home based VCT and p_f _= level of uptake for facility based VCT, using the formula from Hayes et al, a sample size of 472 per group was calculated. The study participants in both groups were offered pretest counseling, HIV antibody testing and post test counseling.

### Instruments

Mixed survey questionnaires for both the baseline and follow up visits were designed as an adaptation from the instruments used by the Voluntary HIV Counseling and Testing Efficacy Study Group [27]. The questionnaires were adapted to make them more context specific. These were translated into the local language of Runyankole, the common language in the study sites and. then back translated into English to ensure commonality and denotative equivalency. The questionnaires were pilot tested, and adjustments were made before the final versions were completed. The questionnaires were interviewer administered. This study was approved by the Institutional Review Board at Mbarara University of Science and Technology.

### Variables

Variables on knowledge and risk perception were operationalized to facilitate statistical analysis using the approach highlighted by Norman et al [28].

#### • Knowledge on transmission of HIV

The respondents were asked 12 questions (items) to assess their knowledge on transmission of HIV. The expected responses were either 'yes', 'no' or 'don't know'. These responses were recoded to 'correct knowledge (1)' for 'yes' and incorrect knowledge (0) for 'no' and 'don't know'. A knowledge score for each respondent was computed by summing the values of each individual item, with a higher score indicating higher levels of knowledge. To conduct the analysis, a respondent was coded to have high knowledge if he or she had 'correct knowledge' on 9 out of the 12 items (correct knowledge on at least 75% of the items). High knowledge was coded '1' and low knowledge coded '0'.

#### • Risk perception

The respondents were asked 3 questions to assess their perceptions about their risk of HIV infection. Four response categories were used: "it almost certainly will not happen", "it could happen", "it probably will happen" and "it almost certainly will happen". These responses were dichotomized. Those who reported 'probably will happen' and "it almost certainly will happen' were coded as high risk perception (1). While those who reported 'it almost certainly will not happen' and 'it could happen' were code as low risk perception (0).

### Analysis

The analysis used the Statistical Analysis Software (SAS) program version 9.1 and Statistical Package for Social Sciences version 12 (SPSS for Windows; SPSS, Chicago, IL, USA). T-tests were used to compare the continuous variables, and two-sided chi-square tests for association were computed to detect differences in categorical variables such as socio-demographic, STI symptoms, HIV knowledge, risk perception, and discrimination related variables between the groups. Probability values (p-values) were calculated at the 0.05 level of significance. In order to explore the degree to which multiple variables were associated with the use of VCT, logistic regression models were run using the forward stepwise variable selection method. The results presented in this paper are derived from only the baseline data.

## Results

### Background characteristics of respondents

A total of 994 respondents were interviewed during the baseline visit. All respondents who were offered either facility based VCT (500) or home based VCT (494) accepted testing and received results. The respondents had a mean age of 32.2 years (SD 10.9) and were mainly female (68 percent). The majority had attended school (78.2 percent) and resided in rural households (62.9 percent). The mean number of years of education completed was 5.5 (SD 4.1). Approximately 61.8 percent were married, 15.7 percent single and 10.7 percent co-habiting.

The background characteristics of the respondents who received VCT through either the facility or home based models are compared in Table 1 below.

**Table 1:** Comparing background characteristics between facility and home based VCT respondents

Characteristic	FB-VCT (N = 499) n (%)	HB-VCT (N = 494) n (%)	P value
Gender			
Male	138 (28)	156 (33)	
Female	351 (72)	326 (67)	0.133+
Age: Mean (SD)	31.8 (11.3)	32.7 (10.7)	0.158λ
Residence			
Trading center	319 (67)	32 (7)	
Rural	153 (33)	450 (93)	<0.0001**
Ever attended school			
Yes	385 (79)	374 (77)	
No	100 (21)	112 (23)	0.351+
Years of education Mean (sd)	5.8 (4.3)	5.2 (4.0)	0.014λ*
Ever married			
Yes	287 (62)	336 (75)	
No	179 (38)	112 (25)	<0.001+**
Monthly income median (mean)	30,000/= (52,100/=)	30,000/= (70,900)	- (0.011)
Age at first sex: Mean (sd)	18.08 (3.3)	18.11 (3.4)	0.916λ
Had sex in past 12 months			
Yes	355 (79)	353 (82)	
No	93 (21)	79 (18)	0.461+
Had VCT in past 12 months			
Yes	142 (29)	81 (17)	
No	345 (71)	399 (83)	<0.001+**

λ t test

+ Chi Square test

** Significant at the 0.001 level

sd = standard deviation

* Significant at the 0.05 level

Similar proportions of male and female respondents received either facility (28% and 72%) or home based (33% and 67%) VCT. The mean age and age at first sex debut of the respondents receiving either option did not differ significantly. The respondents differed with regard to residence, mean years of education completed, marital status and mean monthly income. A larger proportion of the respondents who received facility based VCT resided in trading centers (67%) while those who received home based VCT were from more rural (93%) households (p-value < 0.0001). Similar proportions of the respondents who received either facility (79%) or home based (81%) VCT had had sex in the 12 months prior to the study. However, their VCT experience in 12 months prior to the study differed with a higher proportion of respondents who received the facility based service having accessed VCT previously (p-value < 0.0001).

The respondents were asked to give the reasons for accepting to receive either facility or home based VCT models and these are compared in Table 2.

**Table 2:** Associations between reasons for receiving VCT, presence of STI symptoms, respondents' fear of discrimination, and type of VCT service model

Characteristic	FB-VCT	HB-VCT	OR (95%CI)	aOR (95%CI)
**Reason for receiving VCT (yes)**	**N = 488 n (%)**	**N = 475 n (%)**		
Own past sexual behaviors	5 (1)	6 (1)	0.8 (0.3, 2.7)	
Partners' past sexual behavior	9 (2)	12 (3)	0.7 (0.3, 1.7)	
Taking care of people with HIV/AIDS	7 (1)	15 (3)	0.5 (0.2, 1.1)	
Wanted to know your sero-status	466 (96)	312 (66)	11.6 (7.2, 18.7)	
Planning for the future	12 (2)	17 (4)	0.3 (0.2, 0.4)	
Was asked to volunteer for the study	7 (1)	200 (42)	0.02 (0.01, 0.04)	
**STI symptoms for males (present)**	**N = 128 n (%)**	**N = 141 n (%)**		
Sores on your penis	25 (20)	29 (21)	0.9 (0.3, 2.7)	
Discharge from penis	20 (16)	18 (13)	1.2 (0.6, 2.5)	
Pain or burning on urination	28 (22)	23 (17)	1.4 (0.8, 2.6)	
Itching	39 (31)	29 (21)	1.7 (0.9, 2.9)	
**STI symptom for females (present)**	**N = 351 n (%)**	**N = 326 n (%)**		
Pain or burning on urination	137 (40)	81 (31)	1.5 (1.1, 2.2)	0.63 (0.46, 0.86)
Non-traumatic sores or boils	98 (29)	67 (25)	1.2 (0.8, 1.7)	
Itching	169 (50)	91 (34)	1.9 (1.4, 2.6)	
Abnormal discharge	85 (25)	61 (24)	1.1 (0.7, 1.6)	
**Discrimination related variable (yes)**	**N = 477 n (%)**	**N = 485 n (%)**		
Family will not treat you as a full family member	235 (51)	337 (70)	2.27 (1.74, 2.96)	
Friends will banish you	204 (43)	334 (69)	3.02 (2.32, 3.94)	0.39 (0.26, 0.57)
Community will treat you as an outcast	169 (36)	318 (66)	3.44 (2.64, 4.48)	
Family will not care for you	269 (57)	347 (72)	1.98 (1.51, 1.60)	1.78 (1.22, 2.61)

The respondents who received VCT through the facility based model were more likely to receive the service because they wanted to know their sero-status (OR = 11.6 95% CI = 7.2, 18.7). While those who received home based VCT did so because they wanted to plan for the future (OR = 0.3 95% CI = 0.2, 0.4) or were asked to volunteer for the study (OR = 0.02 95% CI = 0.01, 0.04). The past sexual behavior of the respondents or their partners was not associated with whether they received VCT through either of the models.

### STI symptoms and use of VCT services

The respondents were asked to whether they had STI symptoms in the 6 months prior to the study and these were compared with the use of either model of VCT in Table 2.

Among the males, having had an STI in the 6 month period prior to the study was not associated with use VCT services. The female respondents who received VCT through the facility based model were more likely to have had pain or burning on urination and itching around the vagina (OR = 1.5 95% CI = 1.1, 2.2 and OR = 1.9 95% CI = 1.4, 2.6 respectively).

### Knowledge on HIV transmission and use of VCT services

The respondents were asked whether each of the items shown in Table 3 was a mode of transmission for HIV. The answers to the questions are shown in Table 3.

**Table 3:** Knowledge of respondents on transmission of HIV

Item	Percent answering correctly
Working near someone with the AIDS virus	92.7
Eating food cooked by someone who has the AIDS virus	90.4
Sharing plates, forks, or glasses with someone who has the AIDS virus	76.5
Using public toilets	83.1
Injecting drugs with syringes or needles used by someone who has the AIDS virus	93.6
An infant from his/her mother who has the AIDS virus	83.7
Being coughed or sneezed on by someone who has the AIDS virus	78.4
Having sexual intercourse without a condom with someone who has the AIDS	92.8
A baby breast-fed by his/her mother who has the AIDS virus	87.9
Attending school with a child who has the AIDS virus	91.0
Bites by mosquitoes or other insects who have bitten a person who has the AIDS virus	52.5
Sharing a bed with someone who has the AIDS virus	91.6

Only one item (transmission of the AIDS virus by insects) was answered correctly by less than 70 percent of respondents. The median and mean score for knowledge on transmission of HIV for the whole sample was 11.0 and 10.07 (sd 1.9) respectively The mean score for respondents who received facility based VCT was 9.98 while that for those who received home based VCT was 10.15. This difference in mean score was not statistically significant (t test p-value 0.182). However, when the knowledge was classified into "high" and "low", those who had ever attended school were more likely to have a higher level of knowledge on the transmission of HIV (OR = 2.27 95%CI = 1.60, 3.31). The respondents who received home based VCT had a higher knowledge scores than those who received facility based VCT (OR = 0.7 95% CI = 0.49, 0.97).

### Risk perception and use of VCT services

Respondents' perceptions about their HIV risk were assessed using a series of questions. They perceived themselves at a "high" risk of; getting infected (84%), already having the HIV virus (74%) and eventually developing AIDS (64%). Risk perception was not associated with level of knowledge on HIV transmission (p-value 0.60). The respondents who received facility based VCT were more likely to have a higher personal risk perception than those who received home based VCT (OR = 2.0 95% CI = 1.3, 3.1).

### Discrimination and use of VCT services

The respondents were asked questions to assess their fear of discrimination. The responses are shown in Table 2. Respondents who received home based VCT facility based VCT were more likely to be worried about discrimination (p-value < 0.01).

### Multivariate analysis of factors associated with use of VCT services

The factors that were significantly associated with use of VCT services in the unadjusted analysis were fitted into a multivariable logistic regression model with type of VCT model as the outcome. The results are shown in Table 4.

**Table 4:** Adjusted Odds Ratios from multivariable logistic regression

Predictor	Adjusted OR	95% CI	p-value
Residence (trading center or rural)	0.14	0.07, 0.22	< 0.001
Ever married	2.45	1.66, 3.62	< 0.001
Income categorized into poor/non poor	0.50	0.36, 0.69	< 0.001
Female with an STI	0.63	0.46, 0.86	0.004
Friend will banish you	0.39	0.26, 0.57	< 0.001
Family will not care for you	1.78	1.22, 2.61	0.003

The residence (aOR = 0.14, 95% CI 0.07, 0.22), marital status (aOR = 2.45, 1.66, 3.62), income (aOR = 0.5, 95% CI 0.36, 0.69), females with an STI symptom (aOR = 0.63, 95% CI 0.46, 0.86), fear that that one would be banished by friends if one had AIDS (aOR = 0.39, 95% CI 0.26, 0.57) and fear that one's family would not look after him/her if he or she had AIDS (aOR = 1.78, 95% CI 1.22, 2.61) were independently associated with type of VCT model.

## Discussion

Overall the findings of this study reveal that factors such as residence, marital status, STI symptoms among females, income and fear of discrimination predict uptake of VCT among rural communities. The design of VCT services to improve coverage and utilization should therefore take these factors into consideration.

Consistent with studies conducted elsewhere, the main reason for receiving VCT through either model among the study population was to find out their status as opposed to other reasons such as own or spouse's past sexual behavior [22,29,30]. As demonstrated elsewhere, this may suggest that VCT is feasible and well accepted and may also be a high profile subject among rural communities in south western Uganda [11,30].

Respondents' residence was independently associated with utilization of VCT services. As expected, respondents who resided in trading centers were more likely to seek facility based when compared to those who resided outside these trading centers, in a more rural context. This was largely because health facilities are within the vicinity of trading centers.

The findings show that the proportion of respondents who had previously received VCT in the home based group was lower than that in the facility based group (29% and 17% respectively). These findings are consistent with those from studies done elsewhere which show that ease of access contributes to acceptability between clinic-based and household based VCT [31]. The findings suggest that emphasis should now be placed on improving service coverage by offering VCT services through both models to target the different clusters of rural communities.

The presence of STI symptoms in women was independently associated with the use of VCT services which contrasts findings elsewhere [7]. The women who utilized facility based VCT were more likely to report having an STI symptom. This suggests that women who receive facility based VCT are more willing to disclose the presence of STI symptoms given that their spouses were not likely to be present. In the home setting, fear of repercussions from their spouses in terms of domestic violence constrains the women to reveal existence of any STI related symptoms. The implication here is the provision of VCT in a home setting is likely to lead to failure or a delay in identification of illnesses that may be risk factors for HIV. The provision of home based VCT should therefore be complemented with an assessment of risk factors for HIV particularly STIs and couples encouraged to subsequently seek appropriate care at a health facility.

In contrast to presence of STI symptoms in women, the high self perceived risk among 60% of the respondents was not independently associated with use of either model of VCT. This was consistent with findings elsewhere in rural Uganda [21].

The study findings further show that the respondents were worried about discrimination if they contracted the AIDS virus. Worries about one being banished by the community or his/her family not caring once they have the disease were independently associated with use of VCT services. This is consistent with findings from other studies that showed people that people were uncomfortable with home based VCT because of a likelihood of being suspected to have HIV [11,18]. These findings underscore the importance of confidentiality in a person's decision to use VCT services. The provision of VCT services through either model should include rigorous campaigns against stigma and discrimination while endeavoring to reinforce confidentiality.

### Limitations

The study was confined to two districts in Uganda. However they represent typical "average" districts in a resource limited setting in terms of the extent to which HIV-related practices and objectives are met. Therefore these findings are likely to be similar across Uganda. We also note that this was a cross-sectional study and therefore we cannot define the temporal relationship between the predictor variables and outcome. Further, to assess presence of an STI the respondents were asked to whether they had had any STI symptoms in the 6 months prior to the study. We however note that self reported STI symptoms are not a definitive indicator for sexually transmitted infections.

## Conclusions

The uptake of VCT provided through either the facility or home based models is influenced by client characteristics such as proximity to service delivery points, HIV related symptoms, and fear of discrimination in rural Uganda. Although the home based model is likely to reach populations that previously had little access to VCT there is need to further compare this approach to the facility based model in terms of cost and its effect on reduction of HIV risk behavior.

Given the available empirical evidence, interventions that seek to improve uptake of VCT should provide potential clients with both facility and home based VCT service options within a given setting. The clients are then able to select a model for VCT that best fits their characteristics. This is likely to have positive implications for both service coverage and uptake for different sub-groups within particular communities.

## Competing interests

The authors declare that they have no competing interests.

## Authors' contributions

EMM participated in the conception, design, and implementation of the study, statistical analysis, interpretation and drafting of manuscript. ASA and RG participated in conception, design and implementation of the study. SB participated in the conception, design, interpretation and drafting of the manuscript. All authors read and approved the final manuscript.

## Authors' notes

This article is part of an evaluation of an HIV/AIDS project sponsored by the Istituto Superiore di Sanita of the Republic of Italy. Mulogo is a senior lecturer at Mbarara University of Science and Technology, Aden is the coordinator of the HIV/AIDS project at the Istituto Superiore di Sanita, Guerra is a program director at the Istituto Superiore di Sanita and Sebastian is an associate professor at the Makerere University School of Public Health.

## Pre-publication history

The pre-publication history for this paper can be accessed here:

http://www.biomedcentral.com/1472-6963/11/54/prepub

## References

[B1] UNAIDS 2008 UNAIDS Annual Report: towards universal access 2009 Geneva: UNAIDS/WHO 7 pp. 7

[B2] UNAIDS AIDS Epidemic Update 2005 Geneva: UNAIDS/WHO

[B3] MOH Uganda HIV/AIDS Sero-Behavioural Survey 2004-2005 2006 Kampala: Ministry of Health and ORC Macro

[B4] MOH Ministry of Health PD Health Policy Statement 2006

[B5] Bwambale FM Ssali SN Byaruhanga S Kalyango JN Karamagi CA Voluntary HIV counselling and testing among men in rural western Uganda: Implications for HIV prevention BMC Public Health 2008 263 10.1186/1471-2458-8-263 18664301 PMC2529297

[B6] Shafer AL Biraro S Nakiyingi-Miiro J Kamali A Ssematimba D Ouma J Ojwiya A Hughes P Van der Paal L Whitworth J HIV prevalence and incidence are no longer falling in southwest Uganda: evidence from a rural population cohort 1989-2005 AIDS 2008 22 1641 1649 10.1097/QAD.0b013e32830a7502 18670225

[B7] Wringe A Isingo R Urassa M Maiseli G Manyalla R Changalucha J Mngara J Kalluvya S Zaba B Uptake of HIV voluntary counselling and testing services in rural Tanzania: implications for effective HIV prevention and equitable access to treatment Tropical Medicine and International Health 2008 13 319 327 10.1111/j.1365-3156.2008.02005.x 18397395

[B8] Matovu JK Gray RH Kiwanuka N Kigozi G Wabwire-Mangen F Nalugoda F Serwadda D Sewankambo NK Wawer MJ Repeat Voluntary HIV Counseling and Testing (VCT), Sexual Risk Behavior and HIV Incidence in Rakai, Uganda AIDS Behav 2007 71 78 10.1007/s10461-006-9170-y 17016759

[B9] UNAIDS UNAIDS' Terminology Guidelines 2008

[B10] Bateganya MH Abdulwadud OA Kiene SM Home-based HIV voluntary counseling and testing in developing countries Cohrane Database of Systematic Review 2007 2007 10.1002/14651858.CD006493.pub2 17943913

[B11] Irungu TK Varkey P Cha S Patterson JM HIV voluntary counselling and testing in Nakuru, Kenya: findings from a community survey HIV Medicine 2008 111 117 10.1111/j.1468-1293.2007.00538.x 18257773

[B12] Matovu JK Makumbi F Expanding access to voluntary HIV counselling and testing in sub-Saharan Africa: alternative approaches for improving uptake, 2001-2007 Tropical Medicine and International Health 2007 12 1315 1322 10.1111/j.1365-3156.2007.01923.x 17949401

[B13] WHO Guidance on provider-initiated HIV counselling in Health facilities HIV/AIDS Programme: strenghthening health services to fight HIV/AIDS 2007 WHO

[B14] Service delivery models for HIV counseling and testing http://www.fhi.org/NR/rdonlyres/enjgjg3dojredmsbucesa6ey2i2wbz3ersczzmhjl6pz62ogzln4guycffb4kk2egibf6p5oafwg3k/ModelsofCT2pager122706.pdf

[B15] UNAIDS Epidemic Update: December 2004 2004 Geneva: UNAIDS

[B16] UNAIDS 2008 Report on the global AIDS eoidemic 2008 Geneva: UNAIDS/WHO

[B17] Wolff B Nyanzi B Katongole G Ssesanga D Ruberantwari A Whitworth J Evaluation of a home-based voluntary counselling and testing intervention in rural Uganda Health Policy Plan 2005 20 109 116 10.1093/heapol/czi013 15746219

[B18] Matovu JK Kigozi G Nalugoda F Mangen FW Gray RH The Rakai Project counselling programme experience Tropical Medicine and International Health 2002 7 1064 1067 10.1046/j.1365-3156.2002.00964.x 12460398

[B19] Were W Mermin J Bunnell R Ekwaru JP Kaharuza F Home-based model for HIV voluntary counselling and testing Lancet 2003 361 1569 10.1016/S0140-6736(03)13212-6 12737909

[B20] Okonkwo K Reich K Alabi A Umeike N Nachman S An evaluation of awareness: attitudes and beliefs of pregnant Nigerian women toward Voluntary Counseling and Testing for HIV AIDS PATIENT CARE and STDs 2007 21 252 260 10.1089/apc.2006.0065 17461720

[B21] Matovu JK Gray RH Makumbi F Wawer MJ Serwadda D Kigozi G Sewankambo NK Nalugoda F Voluntary HIV counseling and testing acceptance, sexual risk behavior and HIV incidence in Rakai, Uganda Aids 2005 19 503 511 10.1097/01.aids.0000162339.43310.33 15764856

[B22] Pool R Nyanzi S Whitworth J Attitudes to voluntary counselling and testing for HIV among pregnant women in rural south-west Uganda AIDS CARE 2001 13 605 615 10.1080/09540120120063232 11571007

[B23] Wakholi-Nyanzi B Lara AM Watera C Munderi P Gilks C Grosskurth H The role of HIV testing, counselling, and treatment in coping with HIV/AIDS in Uganda: a qualitative analysis AIDS Care 2009 21 903 908 10.1080/09540120802657498 20024747

[B24] UBOS Statistical Abstract 2009 2009 Kampala: Uganda Bureau of Statistics

[B25] Hayes R Bennett S Simple sample size calculation for cluster-randomized trials Journal of Epidemiology 1999 319 326 10.1093/ije/28.2.319 10342698

[B26] Matovu JKB Kigozi G Nalugoda F Mangen FW Gray RH The Rakai Project counselling programme experience Tropical Medicine and International Health 2002 7 1064 1067 10.1046/j.1365-3156.2002.00964.x 12460398

[B27] AIDSCAP/WHO/CAPS Counseling and Testing Efficacy Study: C & T Baseline Instrument http://www.caps.ucsf.edu/tools/surveys/pdf/baseline%20C&T.pdf

[B28] Norman L Carr R The role of HIV knowledge on HIV-related behaviours: a hierarchial analysis of adults in Trinidad Health Education 2003 103 145 155 10.1108/09654280310472360

[B29] deGraft-Johnson J Paz-Soldan V Kasote A Tsui A HIV Voluntary Counseling and Testing Service Preferences in Rural Malawi Population AIDS and Behavior 2005 9 475 484 10.1007/s10461-005-9018-x 16261266

[B30] Sangiwa G van der Straten A Grinstead O VCTStudyGroup Clients' Perpective of the Role of Voluntary Counseling and Testing in HIV/AIDS Prevention and Care in Dar Es Salaam, Tanzania: The Voluntary Counseling and Testing Efficacy Study AIDS and Behavior 2000 4 35 48 10.1023/A:1009584623712

[B31] Fylkesnes K Siziya S A randomized trial on acceptability of voluntary HIV counseling and testing Tropical Medicine and International Health 2004 9 566 572 10.1111/j.1365-3156.2004.01231.x 15117300

